# Final outcome analysis from the phase II TUXEDO-1 trial of trastuzumab-deruxtecan in HER2-positive breast cancer patients with active brain metastases

**DOI:** 10.1093/neuonc/noae123

**Published:** 2024-07-04

**Authors:** Rupert Bartsch, Anna Sophie Berghoff, Julia Furtner, Maximilian Marhold, Elisabeth Sophie Bergen, Sophie Roider-Schur, Maximilian Johannes Mair, Angelika Martina Starzer, Heidrun Forstner, Beate Rottenmanner, Marie-Bernadette Aretin, Karin Dieckmann, Zsuzsanna Bago-Horvath, Helmuth Haslacher, Georg Widhalm, Aysegül Ilhan-Mutlu, Christoph Minichsdorfer, Thorsten Fuereder, Thomas Szekeres, Leopold Oehler, Birgit Gruenberger, Georg Pfeiler, Christian Singer, Ansgar Weltermann, Luzia Berchtold, Matthias Preusser

**Affiliations:** Department of Medicine I, Division of Oncology, Medical University of Vienna, Vienna, Austria; Department of Medicine I, Division of Oncology, Medical University of Vienna, Vienna, Austria; Research Center for Medical Image Analysis and Artificial Intelligence (MIAAI), Faculty of Medicine and Dentistry, Danube Private University, Krems, Austria; Department of Radiology, Medical University of Vienna, Vienna, Austria; Department of Medicine I, Division of Oncology, Medical University of Vienna, Vienna, Austria; Department of Medicine I, Division of Oncology, Medical University of Vienna, Vienna, Austria; Department of Oncology, St. Joseph’s Hospital, Vienna, Austria; Department of Medicine I, Division of Oncology, Medical University of Vienna, Vienna, Austria; Department of Medicine I, Division of Oncology, Medical University of Vienna, Vienna, Austria; Department of Medicine I, Division of Oncology, Medical University of Vienna, Vienna, Austria; Department of Medicine I, Division of Oncology, Medical University of Vienna, Vienna, Austria; Hospital Pharmacy, Vienna General Hospital and Medical University of Vienna, Vienna, Austria; Department of Radio-Oncology, Medical University of Vienna, Vienna, Austria; Department of Pathology, Medical University of Vienna, Vienna, Austria; Department of Laboratory Medicine, Medical University of Vienna, Vienna, Austria; Department of Neurosurgery, Medical University of Vienna, Vienna, Austria; Department of Medicine I, Division of Oncology, Medical University of Vienna, Vienna, Austria; Department of Medicine I, Division of Oncology, Medical University of Vienna, Vienna, Austria; Department of Medicine I, Division of Oncology, Medical University of Vienna, Vienna, Austria; Department of Laboratory Medicine, Medical University of Vienna, Vienna, Austria; Research Center for Medical Image Analysis and Artificial Intelligence (MIAAI), Faculty of Medicine and Dentistry, Danube Private University, Krems, Austria; Department of Oncology, LKH Wiener Neustadt, Wiener Neustadt, Austria; Department of Obstetrics and Gynaecology, Medical University of Vienna, Vienna, Austria; Department of Obstetrics and Gynaecology, Medical University of Vienna, Vienna, Austria; Department of Medicine 1, Elisabethinen Hospital Linz, Ordensklinikum Linz, Linz, Austria; Institute of Medical Statistics, Center for Medical Data Science, Medical University of Vienna, Vienna, Austria; Department of Medicine I, Division of Oncology, Medical University of Vienna, Vienna, Austria; Department of Medicine I, Division of Oncology, Medical University of Vienna, Vienna, Austria

**Keywords:** brain metastases, breast cancer, HER2-positive, trastuzumab deruxtecan

## Abstract

**Background:**

Brain metastases (BM) are a devastating complication of HER2-positive metastatic breast cancer (BC) and treatment strategies providing optimized local and systemic disease control are urgently required. The antibody-drug conjugate trastuzumab deruxtecan (T-DXd) improved progression-free survival (PFS) and overall survival (OS) over trastuzumab emtansine but data regarding intracranial activity is limited. In the primary outcome analysis of TUXEDO-1, a high intracranial response rate (RR) was reported with T-DXd. Here, we report the final PFS and OS results.

**Patients and Methods:**

TUXEDO-1 accrued adult patients with HER2-positive BC and active BM (newly diagnosed or progressing) without indication for immediate local therapy. The primary endpoint was intracranial RR; secondary endpoints included PFS, OS, safety, quality-of-life (QoL), and neurocognitive function. PFS and OS were estimated with the Kaplan-Meier method and analyzed in the per-protocol population.

**Results:**

At 26.5 months median follow-up, median PFS was 21 months (95% CI: 13.3–n.r.) and median OS was not reached (95% CI: 22.2–n.r.). With longer follow-ups, no new safety signals were observed. The most common grade 3 adverse event was fatigue (20%). Grade 2 interstitial lung disease and a grade 3 symptomatic drop of left-ventricular ejection fraction were observed in one patient each. QoL was maintained over the treatment period.

**Conclusions:**

T-DXd yielded prolonged intra- and extracranial disease control in patients with active HER2-positive BC BM in line with results from the pivotal trials. These results support the concept of antibody-drug-conjugates as systemic therapy for active BM.

**Key Points**

- Brain metastases are a common complication of HER2-positive breast cancer.
- In the TUXEDO-1 trial, the antibody-drug conjugate trastuzumab deruxtecan yielded a high response rate and prolonged progression-free survival and overall survival

**Importance of the Study**

Brain metastases (BM) are commonly observed in HER2-positive metastatic breast cancer (BC) and optimized treatment strategies are urgently required. Recent years have seen a growing interest in systemic therapy. Small-molecule tyrosine-kinase inhibitors yielded clinically relevant activity in patients with newly diagnosed or progressing BM, and tucatinib combined with trastuzumab and capecitabine is currently regarded as the preferred treatment approach. The antibody-drug conjugate (ADC) trastuzumab deruxtecan (T-DXd) provides high clinical activity in patients with pretreated metastatic HER2-positive BC but data regarding activity in BM is limited. The prospective single-arm phase II TUXEDO-1 trial of T-DXd in 15 patients with active BM reported a high intracranial response rate. At the final outcome analysis, median progression-free survival was 21 months and median overall survival was not reached, suggesting prolonged disease control in patients with BM. Importantly, results therefore support the use of T-DXd when clinically indicated even in the presence of active BM.

Brain metastases (BM) increase morbidity and mortality in cancer patients^1^ and breast cancer (BC) is today the second most common cause of BM among solid malignancies.^2,3^ Over the last 2 decades, an increase in BM incidence was reported, mainly attributed to prolonged overall survival (OS) in patients with metastatic HER2-positive disease.^4^ In addition, the brain parenchyma acts as a sanctuary site for cancer cells protected from systemic therapy by the blood-brain barrier (BBB). In line, a numerical increase of BM as the first site of recurrence was reported in patients receiving post-neoadjuvant trastuzumab-emtansine (T-DM1) in the KATHERINE trial, while the risk for extracranial metastases was decreased.^5^

Besides creating a sanctuary in the early disease stage, the BBB was believed to generally prevent the activity of systemic therapy in overt BM as well. Therefore, local treatment (whole-brain radiotherapy [WBRT], stereotactic radiotherapy [SRT], radiosurgery [SRS], and neurosurgery) has long been regarded as the standard of care for established BM.^6,7^ While SRT and SRS provide excellent local disease control in patients with oligometastatic disease, they offer no extracranial activity and concurrent systemic therapy increases the risk for radiation necrosis.^8^ In patients requiring WBRT, prognosis remains poor due to limited activity,^9^ with a more recent study indicating brain-specific progression-free survival (PFS) of 6.5 months with modern radiation techniques^10^; still, neurocognitive decline will eventually occur.^11^ The need for improving intra- and extracranial disease control and treatment tolerability therefore resulted in growing interest in systemic treatment options.

Clinical development of systemic therapy for BC BM initially has focused on HER2-directed tyrosine-kinase inhibitors (TKIs) believed to penetrate the BBB due to their small molecular size.^12^ Based upon the HER2CLIMB trial, the combination of the third-generation TKI tucatinib with the monoclonal HER2-directed antibody trastuzumab and the oral cytotoxic capecitabine (TTC) is regarded as the standard-of-care in patients with active HER2-positive BC BM (ie, newly diagnosed BM or BM progressing after prior local therapy) in the absence of any indication for immediate local therapy.^13,14^ More recently, it was shown that large molecules such as antibody-drug-conjugates (ADCs) yield significant activity in BM, as well as the BBB, is substituted with a more permeable blood-tumor-barrier at the metastatic site.^15–18^ In the TUXEDO-1 trial, the ADC trastuzumab-deruxtecan (T-DXd) yielded an intracranial response rate (RR) of 73.3% in an active BM population in the intention-to-treat population and 78.6% in the per-protocol population (PPP), respectively.^15^ Here, we report the final PFS and overall survival (OS) results as well as updates on QoL and safety from the TUXEDO-1 trial.

## Patients and Methods

TUXEDO-1 is a single-center, single-arm, non-comparative phase II trial evaluating the activity and safety of T-DXd in patients with HER2-positive metastatic BC and active BM defined as newly diagnosed previously untreated BM or BM progressing after prior local therapy. The trial is registered at ClinicalTrials.gov (NCT04752059) and the EU Clinical Trials Register (EudraCT Number: 2020-000981-41). The study was conducted in accordance with the Declarations of Helsinki and Good Clinical Practice and was approved by the local ethics committee (EC number 1359/2020).

### Patients

Details regarding the population for the primary and secondary efficacy endpoints are described in the main publication. In short, TUXEDO-1 included adult patients with histologically confirmed HER2-positive BC and active BM and an Eastern Cooperative Oncology Group (ECOG) performance status <2, and prior exposure to trastuzumab and pertuzumab without indication for immediate local therapy.

### Endpoints and Assessments

The primary endpoint was the rate of best intracranial responses at any radiological assessment after the administration of at least one cycle of T-DXd and intracranial RR was evaluated centrally according to the Response Assessment in Neuro-Oncology criteria in the intention-to-treat (ITT) population. PFS defined as the interval from study inclusion until progression or death and OS defined as the interval from study inclusion until death and safety were key secondary endpoints. Patients without a documented PFS event were censored at the date they were last known to be free of progression. Analysis of PFS and OS was conducted in the PPP.

Sample-size calculation was based on the primary study endpoint.^15^ PFS and OS were estimated with the Kaplan-Meier product limit method and a Cox regression model was used for exploratory analyses of PFS based on ECOG performance status, graded prognostic assessment, hormone-receptor expression, prior T-DM1 therapy, and prior local therapy for BM. All *P-*values are 2-sided.

Safety and tolerability in terms of hematologic and non-hematologic adverse events (AEs) were assessed by the investigators at each visit and graded according to National Cancer Institute Common Terminology Criteria for AEs v5.0. AEs are classified by system organ class and preferred term. Serious AEs (SAEs) were defined according to the International Conference on Harmonization Good Clinical Practice guidelines. All AEs were summarized using frequency counts and percentages. If a patient experienced >1 of any given AE, the patient was only counted once for the most severe grade. All patients who received at least one dose of the study drug were included in the safety population.

QoL and cognitive functioning were assessed with the EORT QLQ-C30 questionnaire, the brain-specific tool (BN20), and the breast-specific tool (BR45) on day 1 of cycles 1, 3, and 5 and every 9 weeks thereafter. A final QoL assessment was conducted at the first survival follow-up at 3 months after end-of-treatment (EOT). Changes from the baseline were analyzed using a linear mixed-effect model. Data were expressed as the mean ± standard error of the mean (SEM). No formal neurocognitive testing was performed and all results concerning global health-related QoL, physical and emotional functioning as well as cognitive functioning are therefore based upon patient-reported outcomes.

Post-progression treatment was captured at the final database lock and is provided for each patient in a descriptive manner.

A biomarker sub-study aimed at investigating changes in the serum levels of serum neuron-specific enolase (sNSE) and serum S100 (sS100) between baseline, cycle 4, and progression as these markers may allow for detection of metastases-induced brain damage.^19–21^ Marker levels were measured as described previously^14^ and are reported as median with range and interquartile range (IQR). The Wilcoxon signed-rank test was used to assess paired differences for each patient. Only patients that had a valid measurement during the treatment phase and upon progression were included in the respective analyses. Statistical tests were performed 2-sided and *P*-values < .05 were considered statistically significant.

Statistical analysis was conducted using R 4.3.1. and IBM SPSS Statistic v28.

## Results

### Patient Characteristics

Between July 2020 and July 2021, a total number of 15 planned patients (14 female, one male) received at least one dose of T-DXd; case report forms for study visits up to data cutoff for the final analysis were collected and data quality controlled with database lock occurring on May 24th, 2023.

Main patient characteristics have been reported previously. In short, 60% had BM progressing after prior local therapy, and 60% had received prior T-DM1. Median age upon inclusion was 69 years (range, 30–76 years), ECOG performance status was 0 in 60% of patients and 40% had neurological symptoms at baseline. Twelve patients had hormone-receptor-positive/HER2-positive disease (80%) and 3 patients had hormone-receptor-negative/HER2-positive disease (20%); brain-only disease was present in 2 participants (13.3%). One patient initially assessed as having parenchymal BM and therefore included was found to have dural metastasis only upon restaging and was therefore included in the primary endpoint analysis in the intention-to-treat population and in the safety population but excluded from secondary endpoint analyses including PFS, OS, QoL, and neurocognitive functioning.

### Efficacy

Median follow-up in the intention-to-treat population was 26.5 months (95% confidence interval [CI] 23.5 months–not reached [n.r.]). Patient characteristics have been reported previously.^14^

**Figure 1.**
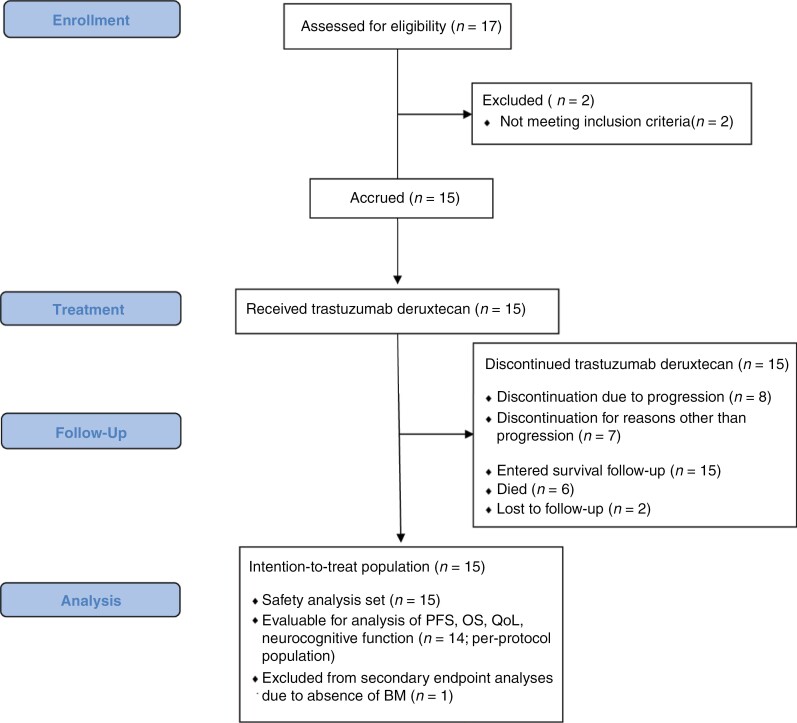
Consort diagram.

At the May 24th, 2023, cutoff, 15 patients had received a total number of 238 cycles of T-DXd (range 4–42 cycles); all patients had discontinued therapy. Reasons for treatment discontinuation were as follows: Disease progression (8 patients; 53.3%; intracranial disease progression as first site of progression 7 patients; synchronous intra- and extracranial disease progression 1 patient), treatment delay longer than allowed by protocol (2 patients; 13.3%), SAEs (2 patients; 13.3%), interstitial lung disease (ILD; 1 patient; 6.7%), left-ventricular ejection fraction drop (1 patient; 6.7%), and patient wish (1 patient; 6.7%). A consolidated standard of reporting trials (CONSORT) diagram is provided in Figure 1.

**Figure 2.**
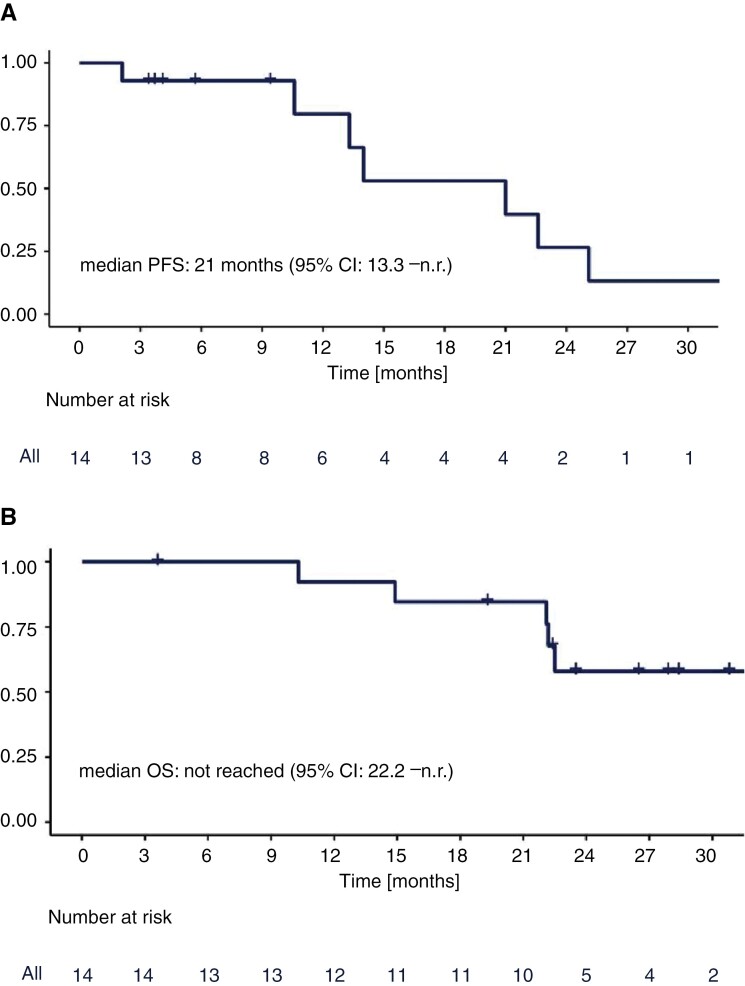
Progression-free survival (A) and overall survival (B).

In the PPP (*n* = 14), median PFS was 21 months (95% CI: 13.3–n.r.; Figure 2A) and irrespective of prior local therapy of BM, prior T-DM1, hormone-receptor status, ECOG performance status, graded prognostic assessment, and dose density; median OS was not reached (95% CI: 22.2–n.r.; Figure 2B). In a post hoc analysis of PFS in the ITT population, the median PFS was 21 months as well. Overall, 6 patients had died: One patient died from urosepsis while on treatment; 5 patients died from disease progression (33.3%; 3/5 died from intracranial disease progression, 1/5 from synchronous intra- and extracranial progression, 1/5 from extracranial progression). Two patients were lost to survival follow-up and therefore censored at the date of last contact. A single patient only received WBRT at the time of intracranial progression; therefore, time-to-WBRT was not evaluable.

### Safety

All 15 patients experienced at least one adverse event (AE; 100%). Most AEs were mild and moderate. Main grade 1/2 hematological toxicities were anemia (46.6%), neutropenia (40.0%), and thrombopenia (6.7%). Grade 1/2 non-hematological AEs observed in more than 2 patients were fatigue (66.7%), nausea (66.7%), upper respiratory tract infections (60.0%), alopecia (46.6%), constipation (46.6%), hypokalemia (40%), vomiting (40%), diarrhea (33.4%), urinary tract infection (33.3%), bone pain (26.7%), thrush (26.6%), blood bilirubin increased (20.0%), dyspnea (20.0%), fall (20.0%), and peripheral sensory neuropathy (20.0%). Regarding alopecia, 2/15 patients (13.3%) experienced grade 2 alopecia. Grade 2 ILD was recorded in a single patient and no case of ILD >grade 2 was observed. Grade 3 AEs related to T-DXd therapy consisted of fatigue (20%; 3 patients), one case of anemia, neutropenia, alanine aminotransferase increase, aspartate aminotransferase increase, diarrhea, dyspnea, left ventricular systolic dysfunction (LVSD), gamma-glutamyltransferase increase, and urinary tract infection, respectively. A summary of all AEs is provided in Table 1. A total of 8 SAEs were recorded in 6 patients (Table 2). Regarding AEs of special interest, grade 2 ILD and grade 3 LVSD were observed in 1 patient each.

**Table 1.** Adverse Events

SOC and PT^1^	*N = 15* ^2^				
Patients with at least one AE^3^	*n* = 15 (100%)^4^				
	Grade 1	Grade 2	Grade 3	Grade 4	Grade 5
	*n* (%)^4^	*n* (%)^4^	*n* (%)^4^	*n* (%)^4^	*n* (%)^4^
*Blood and lymphatic system disorders*					
Anaemia	5 (33.3%)	2 (13.3%)	1 (6.7%)*		
Neutropenia	2 (13.3%)	4 (26.7%)	1 (6.7%)*		
Thrombopenia	1 (6.7%)				
*Cardiac disorders*					
Ejection fraction decreased			1 (6.7%)*		
Palpitations	1 (6.7%)				
*Ear and labyrinth disorders*					
Tinnitus	2 (13.3%)				
Vertigo	2 (13.3%)				
*Eye disorders*					
Extraocular muscle paresis		1 (6.7%)			
*Gastrointestinal disorders*					
Abdominal pain	2 (13.3%)				
Constipation	5 (33.3%)	2 (13.3%)			
Diarrhea	1 (6.7%)	4 (26.7%)	1 (6.7%)*		
Enterocolitis	1 (6.7%)		1 (6.7%)		
Esophageal obstruction		1 (6.7%)			
Flatulence	1 (6.7%)				
Gastritis		1 (6.7%)			
Gastroesophageal reflux disease		1 (6.7%)			
Hemorrhoidal hemorrhage	1 (6.7%)				
Hemorrhoids		1 (6.7%)			
Nausea		10 (66.7%)			
Oral dysesthesia	1 (6.7%)				
Toothache	1 (6.7%)				
Vomiting	1 (6.7%)	5 (33.3%)			
*General disorders and administration site conditions*					
Extravasation	1 (6.7%)				
Fatigue	3 (20.0%)	7 (46.7%)	3 (20.0%)*		
Fever	1 (6.7%)				
Gait disturbance	1 (6.7%)				
Edema face	1 (6.7%)				
Edema limbs	1 (6.7%)	1 (6.7%)			
*Infections and infestations*					
Lung infection		1 (6.7%)			
Laryngitis	1 (6.7%)				
Sepsis					1 (6.7%)
Shingles		2 (13.3%)			
Sinusitis	1 (6.7%)				
Thrush	2 (13.3%)	2 (13.3%)			
Upper respiratory infection	8 (53.3%)	1 (6.7%)			
Urinary Tract Infection		5 (33.3%)	1 (6.7%)		
*Injury, poisoning and procedural complications*					
Fall	2 (13.3%)	1 (6.7%)			
*Investigations*					
Alanine aminotransferase Increased			2 (13.3%) one related^*^		
Aspartate aminotransferase Increased		1 (6.7%)	1 (6.7%)*		
Blood bilirubin increased	3 (20.0%)				
Gamma-glutamyltransferase Increased			2 (13.3%) one related^*^		
Weight gain		1 (6.7%)			
*Metabolism and nutrition disorders*					
Anorexia	2 (13.3%)				
Hypocalcemia	1 (6.7%)		1 (6.7%)		
Hypokalemia	6 (40.0%)				
Hypophosphatemia	1 (6.7%)				
*Musculoskeletal and connective tissue disorders*					
Arthralgia	1 (6.7%)	1 (6.7%)			
Bone Pain	3 (20.0%)	1 (6.7%)			
Muscle Cramp	1 (6.7%)				
Neck Pain	1 (6.7%)				
*Nervous system disorders*					
Dysgeusia	2 (13.3%)				
Headache		2 (13.3%)			
Peripheral sensory neuropathy	2 (13.3%)	1 (6.7%)			
Seizure		1 (6.7%)			
*Psychiatric disorders*					
Anxiety	1 (6.7%)				
Depression		1 (6.7%)			
Insomnia	1 (6.7%)	1 (6.7%)			
Psychosis			1 (6.7%)		
*Respiratory, thoracic, and mediastinal disorders*					
Cough	1 (6.7%)	1 (6.7%)			
Dyspnea		3 (20.0%)	1 (6.7%)^*^		
Epistaxis	1 (6.7%)				
Pneumonitis		1 (6.7%)			
*Skin and subcutaneous tissue disorders*					
Alopecia	5 (33.3%)	2 (13.3%)			
Palmar-plantar erythrodysesthesia syndrome	1 (6.7%)				
Skin and subcutaneous tissue disorders—others: abrasion	1 (6.7%)				
Skin and subcutaneous tissue disorders—others: abscess		2 (13.3%)			
Skin and subcutaneous tissue disorders—others: erythema	1 (6.7%)				
*Vascular disorders*					
Hypertension	1 (6.7%)				
Thromboembolic event	1 (6.7%)	1(6.7%)			

^1^SOC, system organ class; PT, preferred term.

^2^ *N,* number of patients in the safety analysis set.

^3^If a patient experienced >1 of any given AE, the patient is only counted once for the most severe grade.

^4^ *n,* number of patients.

^*^Grade 3/4 AE related to T-DXd.

**Table 2.** Serious Adverse Events^1^

SOC and PT^2^	*N = 15* ^3^
	*n (%)* ^4^
Number of patients with at least one SAE	6 (40%)
Cardiac disorders	
Ejection fraction decreased	1 (6.7%)
General disorders and administration site conditions	
Fatigue	1 (6.7%)
Infections and infestations	
Lung infection	1 (6.7%)
Urinary tract infection	1 (6.7%)
Sepsis	1 (6.7%)
Musculoskeletal and connective tissue disorders	
Pain	1 (6.7%)
Nervous system disorders	
Seizure	1 (6.7%)
Psychiatric disorders	
Psychosis	1 (6.7%)

^1^Serious Adverse Event (SAE): Any adverse event resulting in death, is immediately life-threatening, requires inpatient hospitalization or prolongation of hospitalization, results in persistent or significant disability/incapacity, is a congenital anomaly/birth defect in a child whose parent was exposed to a medicinal product prior to conception or during pregnancy or is considered otherwise medically significant such as important medical events that may not immediately be life threatening or result in death or hospitalization, but jeopardize the subject or require intervention to prevent one of the outcomes listed in the definition above.

^2^SOC, system organ class; PT, preferred term.

^3^ *N,* number of patients in the safety analysis set.

^4^ *n,* number of patients.

A dose reduction by one step was recorded in 5 patients (33.3%) and 2 dose reductions were required in 6 patients (40.0%). Reason for dose reduction were fatigue (5 patients), diarrhea (3 patients), patient wish (2 patients), neutropenia (1 patient), and thrush (1 patient), respectively. Dose delays were observed in 6/238 cycles. Reasons for dose delays were as follows: Fall, upper respiratory infection, LVSD, COVID, urinary tract infection, and fever in one case each.

Six cases of SARS-CoV-2 infections were recorded in the safety population, resulting in 1 serious adverse event due to COVID pneumonia. Therefore, SARS-CoV-2 infections were mild in 5/6 patients and no increased risk for ILD was observed. No case of radiation necrosis was reported (median time from last SRS or SRT to initiation of T-DXd 13.2 months [range 5.7–52.8 months]).

### Quality-of-Life

Among the 14 BM patients eligible for the assessment of health-related QoL and cognitive function based upon patient-reported outcomes, all 14 evaluable patients completed ≥1 assessment. Global health status was maintained over the entire treatment period in the PPP; comparable results were observed regarding emotional and physical functioning as well as cognitive functioning. In patients who had documented disease progression and at least one QoL assessment at or after EOT (*n* = 7), a significant drop in global QoL was observed upon progression (*P* = .036) (Supplementary Figure 1).

### Further Treatment

In the PPP, one patient discontinuing T-DXd due to interruption longer than allowed received best supportive care only. One patient discontinuing T-DXd due to a LVSD continued T-DXd off-study upon recovery of systolic function. Two patients were lost to follow-up upon progression. SRS was administered upon intracranial progression in 3 patients, with 2/3 continuing T-DXd and 1/3 single-agent trastuzumab in the absence of extracranial disease.

In 5 patients TTC was the immediate next treatment line after T-DXd. Primary progression occurred in 2/5 patient, 1/5 patients had intracranial progression after 5 months, received SRS, and continued TTC up until data cutoff without any further intra- or extracranial progression event. In one patient, TTC was initiated in November 2021 upon discontinuation of T-DXd due to ILD and treatment continued up until the next progression event for a total duration of 14.6 months. Finally, in 1 patient, TTC therapy has been ongoing since December 2022. Median duration of TTC was 3.3 months (range 2.3 + −14.6 months).

### Biomarker Analysis

**Figure 3.**
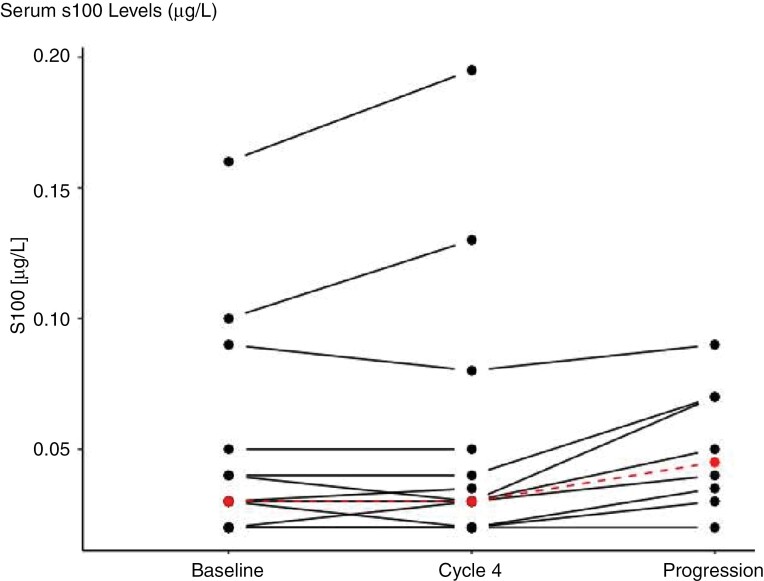
Serum S100 levels (µg/L) at baseline, cycle 4, and progression.

sNSE and sS100 levels were assessed in a total of 71 blood samples (cycles 1, 4, and EOT). Matched samples from all timepoints were available in 8 patients. Median sNSE levels were 10.6 ng/mL (*n* = 13; range 7.3–44.9 ng/mL; IQR 8.7–12.2) at baseline, 10.5 ng/mL (*n* = 14; range 5.9–13.1 ng/mL; IQR 7.1–13.4) at cycle 4, and 10.6 ng/mL (*n* = 8; range 7.7–17.5 ng/mL; IQR 8.4–10.9) upon disease progression respectively (Wilcoxon signed-rank test; *P* = .4). Regarding s100 levels, respective numbers were 0.03 ng/mL (*n* = 14; range 0.02–0.27 ng/mL; IQR 0.03–0.06) at baseline, 0.03 ng/mL (*n* = 14; range 0.02–0.20 ng/mL; IQR 0.02–0.05) at cycle 4. Upon progression, a significant increase of s100 levels was observed (*n* = 8; 0.05 ng/mL; range 0.02–0.09 ng/mL; IQR 0.03–0.07; Wilcoxon signed-rank test; *P* = .02; Figure 3).

## Discussion

The phase II TUXEDO-1 trial was designed to evaluate the activity and safety of T-DXd as systemic therapy in metastatic HER2-positive BC patients with active BM in the absence of any immediate indication for local therapy. The main outcome analysis has already been reported and TUXEDO-1 has met the primary study endpoint with an intracranial response rate of 73.7% as measured by Response Assessment in Neuro-Oncology -BM criteria in the ITT population.^15^ Here, key secondary endpoints of progression-free and overall survival are presented. At the final database lock with a median follow-up of 26.5 months, all patients had discontinued study treatment; median PFS was 21 months, and median OS was not reached. While derived from a single-arm, single-center phase II trial, long-term outcomes of TUXEDO-1 support the role of ADCs as systemic therapy for active BM and need to be discussed in the light of results of other studies evaluating systemic therapy in HER2-positive BC BM.

The activity of small-molecule HER2-targeting TKIs—both as upfront systemic therapy and in progressive BM—is well established.^13,22–24^ In the randomized HER2CLIMB trial, tucatinib, when added to trastuzumab and capecitabine, prolonged PFS from 4.1 to 9.5 months in the active BM population (HR 0.36; 95% CI: 0.22–0.57) and OS was improved from 11.6 to 20.7 months (HR 0.49; 95% CI: 0.30–0.80) in this patient subset; TTC yielded an intracranial RR of 47.3% (95% CI: 33.7%–61.2%) in patients with measurable disease (*n* = 75).^13^ While the ESMO/ABC guidelines therefore list TTC as the preferred option in patients with active HER2-positive BC BM, activity of ADCs has been recently documented as well.

In the phase IIIb KAMILLA trial T-DM1, the first ADC approved for the treatment of metastatic HER2-positive BC, yielded an intracranial response rate of 49.3% (33/67; 95% CI: 36.9–61.8) in the subset of patients with measurable BM without prior radiotherapy. Median OS was 18.9 months (95% CI: 17.1–21.3) in patients with baseline BM.^25^ Findings of KAMILA therefore compare favorably with results of HER2CLIMB and suggest clinical activity of ADCs in BM in principle. The phase III DESTINY-Breast03 established T-DXd as the current second-line standard in HER2-positive mBC. Here, the superiority of T-DXd over T-DM1 was maintained in the subset of patients with BM at baseline (median OS 25.1 months [T-DM1] vs. not reached [T-DXd]; HR 0.54; 95% CI: 0.29–1.03).^26^ The advantage of T-DXd over T-DM1 is based upon its specific pharmacological properties resulting in a bystander effect targeting the microenvironment.^27^ Given the close interaction of tumor cells with autochthonous brain cells,^12^ this bystander effect is of specific interest in BM. In line, a recent pooled analysis of outcomes of patients with baseline BM accrued to the DESTINY-Breast01, 02, and 03 trials, reported an intracranial response rate of 44.5% in the subset of patients with previously untreated asymptomatic (ie, active) BM (*n* = 44); here, CNS PFS was 18.5 months (95% CI: 13.6–23.3 months).^28^ With all limitations of cross-trial comparisons, long-term outcomes of TUXEDO-1 therefore appear comparable with findings of this post hoc analyses from the pivotal T-DXd trials. Together with favorable results from the phase II DEBBRAH study,^16^ results, therefore, support T-DXd as the second-line standard in HER2-positive mBC irrespective of the presence of BM.

Regarding toxicity, no new safety signals were observed. While an increased risk for radiation necrosis linked to ADC treatment after SRS or SRT was suggested,^8,29^ not a single case was observed in TUXEDO-1. This is probably due to the relatively long time period from the last local intervention until the initiation of T-DXd with none of the patients having an interval of less than 3 months. The small sample size may also have led to an underestimation of the true radiation necrosis risk. Finally, radiation necrosis incidence was mainly assessed in patients receiving T-DM1 to date and risk may be lower with T-DXd. Global QoL and cognitive function were maintained over the duration of treatment. Therefore, data suggest T-DXd to be safe in this population.

Despite the unprecedented activity of T-DXd in metastatic HER2-positive BC, patients will ultimately progress and will require further treatment. Five patients (33.3%) received TTC as the immediate next treatment line; in a post hoc analysis conducted in this population, the median treatment duration was 3.3 months (range 2.3 + −14.6 months). While disease control in patients receiving TTC was therefore apparently shorter compared with the HER2CLIMB trial, it must be remembered that this was likely a more heavily pretreated patient sample. With all limitations of a non-prespecified post hoc analysis, data therefore suggest that sequential systemic therapy of active HER2-positive BM may be possible but combined modality treatment encompassing local therapy is often required. In an exploratory biomarker analysis, we observed a significant increase in sS100 levels upon intracranial progression, suggesting a potential role for treatment monitoring. As this analysis is based on samples from 8 patients only, further evaluation in larger studies is required.

The single-arm, single-center design, and the small sample size are obvious limitations of TUXEDO-1, bearing the risk of an inclusion bias. While this may be less relevant for the analysis of intracranial RR, it may impact long-term endpoints such as PFS and OS to a larger extent and absolute numbers must therefore be interpreted with caution. Despite these limitations, long-term outcomes of TUXEDO-1 support the hypothesis that despite its large molecular size, T-DXd yields clinically relevant activity in active BM from HER2-positive BC and allows for prolonged intra- and extracranial disease control with acceptable tolerability and maintained QoL function. In summary, these findings suggest that T-DXd is a valid alternative to TKIs for the treatment of active BM and may be preferred in the presence of extensive and/or symptomatic extracranial disease.

## Supplementary material

Supplementary material is available online at *Neuro-Oncology* (https://academic.oup.com/neuro-oncology).

noae123_suppl_Supplementary_Figures_1

## Data Availability

Upon request, individual participant data that underlie the results reported in this article will be made available after deidentifiction. In addition, the study protocol and the informed consent form will be available. Data will be available immediately following publication for an indefinite period. Data will be made available to researchers whose proposed use of the data has been approved by an independent review committee to achieve the aims in the approved proposal. All proposals should be directed to the corresponding author, and data requestors will need to sign a data access agreement.
