# Supplementary material for: Final outcome analysis from the phase II TUXEDO-1 trial of trastuzumab-deruxtecan in HER2-positive breast cancer patients with active brain metastases
Source: Neuro Oncol. 2024 Jul 4;26(12):2305–15. doi: 10.1093/neuonc/noae123 (PMC11630562; doi:10.1093/neuonc/noae123)
Supplement: noae123_suppl_Supplementary_Figures_1 [file noae123_suppl_supplementary_figures_1.docx]

**SUPPLEMENTARY FIGURES**

**Supplementary Figure 1**

**Supplementary Figure 1a: Global health-related quality-of-life**


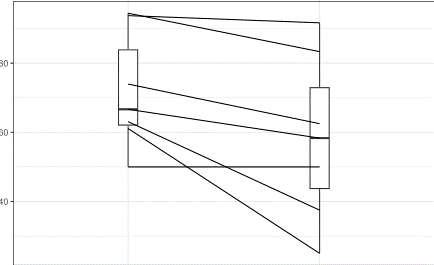


Baseline Progression

Changes in health-related quality of life from baseline to progression

**Supplementary Figure 1b: Cognitive function**


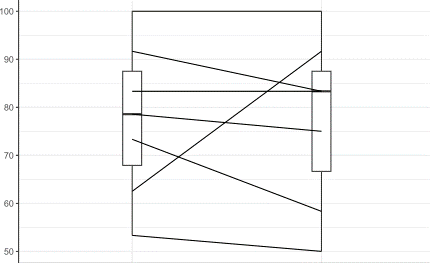


Baseline Progression

Changes in cognitive function from baseline to progression
